# P-2248. Modeling the Economic Impact of Different Testing Strategies for Meningitis/Encephalitis in Adult Patients from a US Hospital Perspective

**DOI:** 10.1093/ofid/ofae631.2401

**Published:** 2025-01-29

**Authors:** Rodrigo Hasbun, Kyle Hueth, Glaucia Paranhos-Baccala, Tristan T Timbrook, Lohit Korrapati, Catherine Regan, Adrienne Kwok, Noam Kirson

**Affiliations:** UT Health Mc Govern Medical School, Houston, Texas; bioMérieux, Salt Lake City, Utah; bioMérieux, Salt Lake City, Utah; bioMerieux, Salt Lake City, Utah; Analysis Group, San Francisco, California; Analysis Group, San Francisco, California; Analysis Group, San Francisco, California; Analysis Group, Inc., Boston, Massachusetts

## Abstract

**Background:**

Meningitis/encephalitis (ME) is a life-threatening disease whose severity and trajectory vary by etiology. Many times, patients with suspected ME are unnecessarily hospitalized and treated with empiric therapy as a precaution while clinicians determine etiology. ME diagnosis and treatment algorithms vary across hospitals, with some using a battery of rapid diagnostic tests (RDTs) or syndromic testing to inform diagnosis and clinical decisions and others relying on bacterial culture and send-out tests. We modeled the economic impact of 4 ME testing strategies from a US hospital perspective.

**Figure:**
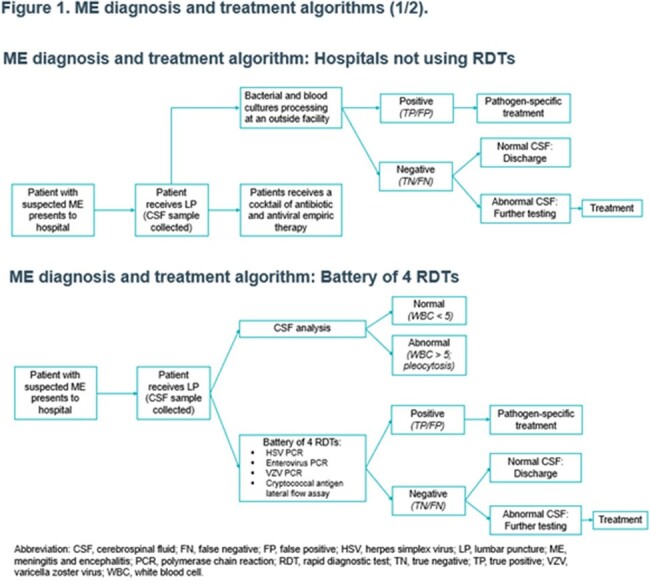

**Methods:**

A simulation model was used to generate a large sample of 20,000 suspected ME cases based on known distributions of ME etiology and cerebrospinal fluid (CSF) analysis results. The model simulated diagnosis, clinical decisions, and resource use and costs to hospitals in 4 testing scenarios: (1) no RDTs, (2) a battery of 4 RDTs for HSV, enterovirus, VZV, and *C. neoformans/gattii*, (3) BIOFIRE^®^ FILMARRAY^®^ ME panel alone [syndromic testing], and (4) syndromic testing in conjunction with RDTs for HSV and *C. neoformans/gattii* (Figures 1-2). Mean inpatient resource use (i.e., hospital stay and antimicrobial use) and cost per suspected ME case were estimated. Common costs across testing scenarios were excluded. Sensitivity analyses were conducted.

**Figure:**
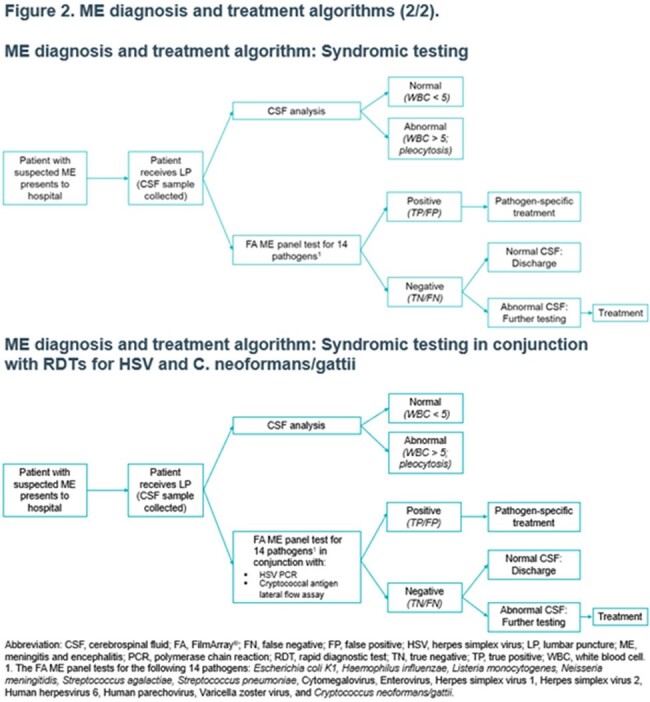

**Results:**

The mean (95% CI) cost per suspected ME case was estimated at $19,337 ($19,150-$19,525) with no RDT use, $16,412 ($16,226-$16,598) with the battery of 4 RDTs, $15,465 ($15,285-$15,644) with syndromic testing, and $15,720 ($15,536-$15,903) under conjunction testing. Cost savings were driven primarily by reductions in hospital stay due to reduced time to correct pathogen identification. Performance characteristics of RDTs/syndromic test and time to correct pathogen identification had the largest impact on modeled costs for strategies that include RDT/syndromic testing.

**Conclusion:**

Using either the battery of 4 RDTs or syndromic testing would result in cost savings for hospitals currently not using RDTs in ME diagnosis and treatment. Syndromic testing would yield even higher savings than the battery of 4 RDTs by further reducing unnecessary hospital stays and inappropriate antimicrobial use.

**Disclosures:**

Rodrigo Hasbun, MD MPH FIDSA, Biomeriaux: Grant/Research Support|Biomeriaux: Honoraria Kyle Hueth, MS, MLS(ASCP)SC, bioMérieux: Stocks/Bonds (Public Company) Glaucia Paranhos-Baccala, PhD, bioMérieux: Stocks/Bonds (Public Company) Tristan T. Timbrook, PharmD, bioMérieux: Stocks/Bonds (Public Company) Lohit Korrapati, MBA, bioMérieux: Advisor/Consultant Catherine Regan, MPH, bioMérieux: Advisor/Consultant Adrienne Kwok, MPH, bioMérieux: Advisor/Consultant Noam Kirson, PhD, bioMérieux: Advisor/Consultant

